# Body site microbiota of Magellanic and king penguins inhabiting the Strait of Magellan follow species-specific patterns

**DOI:** 10.7717/peerj.16290

**Published:** 2023-11-02

**Authors:** Manuel Ochoa-Sánchez, Eliana Paola Acuña Gomez, Lucila Moreno, Claudio A. Moraga, Katherine Gaete, Luis E. Eguiarte, Valeria Souza

**Affiliations:** 1 Instituto de Ecología, Universidad Nacional Autónoma de México, CDMX, Mexico; 2 Centro de Estudios del Cuaternario de Fuego, Patagonia y Antártica (CEQUA), Punta Arenas, Chile; 3 Posgrado en Ciencias Biológicas, Universidad Nacional Autónoma de México, Ciudad de México, México; 4 Departamento de Zoología, Facultad de Ciencias Naturales y Oceanográficas, Universidad de Concepción, Concepción, Chile

**Keywords:** *Psychrobacter*, Seabird microbiota, Ecological drift, Marine host microbiome, Marine sentinel microbiome, Microbial ecology, Metabarcoding, *Clostridum sensu stricto*

## Abstract

Animal hosts live in continuous interaction with bacterial partners, yet we still lack a clear understanding of the ecological drivers of animal-associated bacteria, particularly in seabirds. Here, we investigated the effect of body site in the structure and diversity of bacterial communities of two seabirds in the Strait of Magellan: the Magellanic penguin (*Spheniscus magellanicus*) and the king penguin (*Aptenodytes patagonicus*). We used 16S rRNA gene sequencing to profile bacterial communities associated with body sites (chest, back, foot) of both penguins and the nest soil of Magellanic penguin. Taxonomic composition showed that Moraxellaceae family (specifically *Psychrobacter*) had the highest relative abundance across body sites in both penguin species, whereas Micrococacceae had the highest relative abundance in nest soil. We were able to detect a bacterial core among 90% of all samples, which consisted of *Clostridium sensu stricto* and Micrococcacea taxa. Further, the king penguin had its own bacterial core across its body sites, where *Psychrobacter* and *Corynebacterium* were the most prevalent taxa. Microbial alpha diversity across penguin body sites was similar in most comparisons, yet we found subtle differences between foot and chest body sites of king penguins. Body site microbiota composition differed across king penguin body sites, whereas it remained similar across Magellanic penguin body sites. Interestingly, all Magellanic penguin body site microbiota composition differed from nest soil microbiota. Finally, bacterial abundance in penguin body sites fit well under a neutral community model, particularly in the king penguin, highlighting the role of stochastic process and ecological drift in microbiota assembly of penguin body sites. Our results represent the first report of body site bacterial communities in seabirds specialized in subaquatic foraging. Thus, we believe it represents useful baseline information that could serve for long-term comparisons that use marine host microbiota to survey ocean health.

## Introduction

The skin is the first barrier between animals and their environment (Ross, Rodrigues Hoffmann & Neufeld, 2019). It harbors a diverse microbial community including viruses, fungi, bacteria, and archaea that seems to be host-specific (Colston & Jackson, 2016). Skin bacterial composition is driven by microenvironmental heterogeneity (Costello et al., 2009), environmental surroundings (Song et al., 2013), and host-specific factors such as diet (Divya et al., 2015) and age (Capone et al., 2011). Furthermore, skin microbiota composition could be relevant for host fitness; for example, in amphibians certain skin bacteria can prevent fungal diseases (Rebollar et al., 2016; Rebollar et al., 2019). Most studies have been conducted on humans, domestic animals, and amphibians, whereas the skin or feathers of birds, especially seabirds, have received less attention (Ross, Rodrigues Hoffmann & Neufeld, 2019).

Feathers are a critical trait that protects birds from environmental stressors such as UV radiation, overheating, and cold. Hence, feathers are a key adaptation in birds’ evolutionary history that has enabled them to colonize different environments throughout the world (Xu et al., 2014). Feathers are considered as a harsh microenvironment, due to their constant desiccation and poor nutrient content, yet they are routinely colonized by bacteria (microbiota, hereafter) (Ross, Rodrigues Hoffmann & Neufeld, 2019). For example, the feathers and skin of continental birds are routinely colonized by soil microbes (Shawkey et al., 2005; Saag et al., 2011; Van Veelen, Falcao Salles & Tieleman, 2017). However, there are other relevant factors influencing feathers and skin microbiota, like sex (Gunderson, Forsyth & Swaddle, 2009), species-specific (Engel et al., 2018), antagonistic interactions (Javurkova et al., 2019), social habits (Engel et al., 2020), body site microenvironmental heterogeneity (Leclaire et al., 2019; Leclaire et al., 2023), preening (Shawkey, Pillai & Hill, 2003), and prolonged exposure to solar radiation (Saranathan & Burtt, 2007; Graves et al., 2020).

The examples reviewed above come from continental bird species, whereas the skin and feather microbiota of seabirds remains poorly studied. Recent research on the feather microbiota of seabirds shows that different factors influence feather microbiota composition (Pearce et al., 2017; Leclaire et al., 2019; Leclaire et al., 2023). In the Leach’s storm petrel (*Oceanodroma leucorhoa*), feather and skin microbial communities are driven by sex and body-site microenvironmental differences, whereas environmental microbes have a minor influence (Pearce et al., 2017). In black-legged kittiwakes (*Rissa tridactyla*) and blue petrels (*Halobaena caerulea*), microbial composition differs both across body sites and between individuals (Leclaire et al., 2019; Leclaire et al., 2023). Although these seabirds are in contact with the marine environment, they are not specialized in subaquatic foraging and swimming, like penguins (De Roy, Jones & Cornthwaite, 2022). Subaquatic foraging and its associated changes in pressure likely exert an additional selective filter on microbes colonizing penguin feathers and skin. Hence, any microbe that might persist on penguin body sites should be able to cope with fluctuations in depth pressure between dives, as well as environmental (*e.g.*, solar and wind exposure) and biological factors (*e.g.*, preening).

Penguin microbiota research has focused mostly on feces, while penguin feather and skin microbiota remain largely unknown (Ochoa-Sánchez et al., 2023). Since feather microbiota could be influenced by its environment, any environmental fluctuation must be taken into consideration when addressing this topic. In penguins, reproductive ecological strategies are an important source of body environmental exposure. For example, penguins may nest on open land, under bushes, or in burrows (De Roy, Jones & Cornthwaite, 2022). Thus, nesting strategy determines body site exposure when on land, such that penguins that nest on open (*e.g.*, king penguin, *Aptenodytes patagonicus*) have the greatest environmental exposure. This is in contrast with penguins that nest in burrows, which are sheltered from prolonged environmental exposure (*e.g.*, Magellanic penguin, *Spheniscus magellanicus*) (Boersma et al., 2013; Bost et al., 2013). We hypothesize that differences in nesting strategies could be related to body site microbiota diversity and composition. For instance, penguins that are under constant environmental exposure (*i.e.*, those that nest on open land) might have more microenvironmental heterogeneity across their body sites (*e.g.*, back influenced by wind and solar radiation), which could result in body site-specific microbiotas (Grice et al., 2009). In contrast, burrow-nesting penguins have intermittent environmental exposure, which could attenuate body site microenvironmental differentiation. Furthermore, soil microbiota from the burrow is likely to influence the feather and skin microbiota of penguins given the direct contact between these surfaces that occurs within the burrow (Kilgas et al., 2012; Van Veelen, Falcao Salles & Tieleman, 2017).

Despite differences in breeding ecological strategies between penguin species, all penguins experience remarkable environmental variability, both abiotic (*e.g.*, solar radiation, marine influence, wind) and biotic (*e.g.*, preen oil application). These in turn could result in body site microbiota driven by neutral processes, where environmental variability enhances the role of stochastic process in microbial community shifts (Costello et al., 2012; Sieber et al., 2019). Microbiota assembly driven by neutral processes should apply more to penguins that nest on open land since their environmental exposure is stronger. Body site microbiota influenced by neutral processes might be a composite effect of the constant colonization of microbes from different environmental sources and host physiological filters. In contrast, burrow-nesting penguins experience less environmental exposure, which could decrease the relevance of neutral processes.

In this study, we investigated the effect of body site on the structure and diversity of feather and skin microbiota of two seabirds, the Magellanic and king penguins. In addition, we addressed the potential influence that nest soil microbiota could have on Magellanic penguin body site microbiota. We did not include king penguin nest samples because nesting occurs on open land, specifically in a brood patch located between the bird legs (Bost et al., 2013). We propose that these results will be useful as baseline data that could serve for long-term comparisons that address the ongoing effects of climate change and environmental degradation in southern marine ecosystems. We suggest that penguin body site microbiota could be used to address marine ecosystem quality at the microbial level, expanding their role as marine sentinels (Boersma, 2008). We evaluated two sets of hypotheses. First, at the intraspecific level, which refers to variation between body site microbiota within each species due to differential environmental exposure. We predicted that bacterial communities of penguin body sites will differ in their composition and diversity. Second, at the interspecific level, which involves variation among body site microbiota between species due to differences in breeding ecological strategies. We predicted differences in body site microbiota diversity and composition between penguin species. To accomplish this, we used high-throughput sequencing of the V3–V4 region of the 16S rRNA gene to characterize the microbiota inhabiting feathers (*i.e.,* back and chest) and skin (*i.e.,* feet) of both species of penguins, as well as Magellanic penguin nest soil.

## Materials and Methods

### Bioethical statement

The penguins were captured and contained by a qualified team of at least three operators following bioethical guidelines of the Comité de Ética, Bioética y Bioseguridad, Universidad de Concepción (protocol number CEBB 1081-2021). The sampling was performed following approved guidelines by Subsecretaría de Pesca y Acuicultura (resolutions E-2021-531; RES. EX. 3315), which approved the sampling of up to 45 birds per locality per year. The permits specified several localities in the Strait of Magellan, including Contramaestre Island and Tierra del Fuego. Given that our goal was feathers and foot skin microbiota, our sampling did not produce any significant physical damage to birds, that is, no penguin was harmed nor killed in this study. After sample collection penguins were released into their habitat.

### Sample collection, storage, and processing

**Figure 1:**
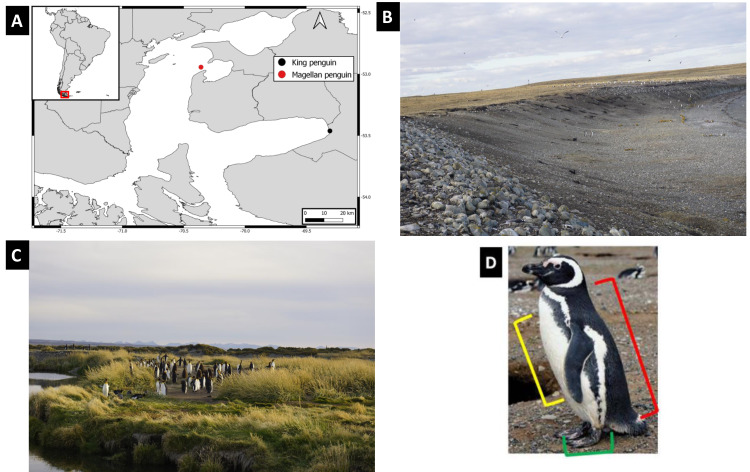
Study localities characteristics, geographic location and body sites considered. (A) Penguin sampling sites, red mark points to Contramaestre Island, whereas black mark points to Pingüino Rey Natural Reserve. Top left inset show localities position at a continental scale. (B) Photo of Contramaestre Island with the colony of Magellanic penguin *Spheniscus magellanicus*. (C) Photo of Pingüino Rey Natural Reserve with the king Penguin, *Aptenodytes patagonicus* colony. (D) Body sites sampled (illustrated with a *Spheniscus magellanicus*) in this study, red bracket indicates the back, yellow indicates chest, while green points the feet. Image credit: Manuel Ochoa-Sánchez.

Penguin body site samples were collected during two periods in 2021, at two remote localities in the Strait of Magellan (Fig. 1): Contramaestre Island colony (November) and Pingüino Rey Natural Reserve in Tierra del Fuego Island (December). Contramaestre Island is an important Magellanic penguin colony in the Strait of Magellan, with ca. 13,000 breeding pairs (CEQUA, 2018). This Island is in the northeastern area of the Strait of Magellan near Gente Grande Bay in Tierra del Fuego Island, Chile. The Pingüino Rey Natural Reserve is a small, recent colony that was established ca. 2010 (Kusch & Marín, 2012). It protects ca. 160 king penguins in the only inland breeding colony of the species, located on the shore of the Inutil Bay on the island of Tierra del Fuego. Sampling was conducted on 11 adult Magellanic penguins walking along the beach and nine Magellanic penguin active nests on Contramaestre Island. In Pingüino Rey Natural Reserve eight adult king penguins were sampled (Table 1).

**Table 1:** Number, type, and body site sampled for microbiota per penguin species.

Species	Body site	Sample type	N
*Spheniscus magellanicus*	Chest	Feather	10
	Back	Swab	11
	Feet	Swab	10
	Nest	Swab	9
*Aptenodytes patagonicus*	Chest	Feather	8
	Back	Swab	8
	Feet	Swab	8

To prevent penguin feathers and feet contamination by the researchers, penguin manipulation was performed with sterile nitrile gloves and clinical diving suits that were changed prior to manipulate a new individual bird. The initial goal was to use feathers for sampling, but as sampling proceeded, we noted that this increased animal stress, so we changed to sterile swabs (Puritan, Guilford, ME, USA). Thus, chest feathers and back swabs were sampled. For sampling using swabs, we rubbed each penguin’s body site five times with three swabs. For the nest soil sample, active nests were selected, *i.e.,* nests with penguins inside or observed to be occupied. From each nest, and at 30 cm from the nest entrance, three soil swabs were collected. All swabs and feather samples were placed in 2 ml cryotubes and stored in a cold container for less than 8 h and then transferred to a liquid nitrogen tank. Later, they were kept at −80 °C at the Molecular Genetics Laboratory in CEQUA, Punta Arenas, Chile facilities.

### DNA extraction and sequencing

DNA extractions were carried out in April 2022, five months after sample collection. We used two swabs from penguin body sites (*i.e.,* back and foot), one swab from nest soil, and one feather from the penguins’ chest for DNA extraction. To extract DNA, we used FastDNA™ SPIN Kit for Soil (MPBio, Santa Ana, CA, USA) following manufacturer instructions with an initial incubation step at 56 °C for 20 min, followed by agitation with Fast Prep at 6 m/s for 40 s. In addition, we performed blank extractions from kit reagents to amplify the 16S rRNA gene to detect potential contaminants and inspected the results in an agarose gel (Salter et al., 2014). DNA extractions were concentrated using a SpeedVac Thermo Savant (model DNA 120-230; Thermo Fisher Scientific, Waltham, MA, USA) for 30 min with standard conditions, which reduced volume to half (*i.e.,* from 100 to 50 µl). We sent 15 µl of concentrated DNA to Macrogen (Seoul, South Korea) to perform amplification of the hypervariable region V3–V4 of the 16S gene using the universal primers 341F-805R (Klindworth et al., 2013). Library preparation and paired-end (2 × 300 pb) sequencing were performed on the Illumina MiSeq sequencing platform at Macrogen (Seoul, South Korea).

### Inference of amplicon sequence variants

To describe amplicon sequence variants (ASVs), sequences were processed using DADA2 v1.18.0 (Callahan et al., 2016a), following the DADA2 workflow tutorial (Callahan et al., 2016b) in R v4.1.3 (R Core Team, 2022). Forward and reverse reads were trimmed at the first 20 nucleotides to remove primer sequences. Then, both forward and reverse reads were truncated at positions 290 and 250, respectively. Ambiguous bases were not allowed, and a maximum of two expected errors was set. Subsequent steps, including error rates learning, dereplication, denoising, and merging of paired reads were performed using default parameters (Callahan et al., 2016b). Taxonomic assignment was performed with the naïve Bayesian classifier (Wang et al., 2007), natively implemented in DADA2 using the Silva v138 database as a reference (Quast et al., 2013). A phylogenetic tree was generated in R v4.1.3 following Callahan et al. (2016b). First, non-chimeric sequences were aligned and used to perform a neighbor-joining tree (NJ). Then, the NJ tree was used as a template to estimate a Maximum Likelihood phylogeny. Alignment and phylogenetic trees were created with DECIPHER v2.22 (Wright, 2016) and phangorn v2.9 (Schliep, 2011).

We used the frequency method of the decontam package to filter sequences flagged as contaminants (Davis et al., 2018). Sequences assigned to plant chloroplasts and mitochondria were discarded. Sequences with low prevalence (*i.e.,* only detected in one sample) were also discarded. Filtered reads were assessed for sufficient depth coverage with rarefaction plots (Files S1 and S2). Both species samples reached the plateau at 20,000 sequences. Thus, we rarefied samples to the lowest sample size, which was 30,100 sequences. Rarefying has been criticized as it discards sequences arbitrarily (McMurdie & Holmes, 2014). However, variance-stabilizing methods that allow the usage of non-rarefied samples distort ecological distance metrics (McKnight et al., 2019). Sample rarefying is an accurate approach to performing distance-based community comparisons, while variance stabilizing methods are recommended to conduct differential abundance tests (McKnight et al., 2019). Since we did not perform any differential abundance analysis, we performed all subsequent analyses with rarefied samples.

### DNA yield analysis

To determine if sampling differences produced differences in DNA concentration, we compared the final DNA yield per sample reported by Macrogen before PCR amplification. First, we explored variance homogeneity with the Bartlett test. Based on these results we compare global DNA yield among sample types with Kruskal–Wallis test. Upon significant results, we performed a paired Wilcoxon test to detect which specific sample types differed.

### Community analysis

All statistical analyses were conducted in R v4.1.3 and RStudio v1.3 (R Core Team, 2022; RStudio, Inc., 2020). We used the microbiome package v1.12.0 to produce heatmaps with the ten most abundant bacterial families and to detect the most abundant taxa across penguin sample types (Lahti & Shetty, 2017). To visualize unique ASVs across penguin sample types, we used up set plots with the UpSetR package v1.4.0 (Conway, Lex & Gehlenborg, 2017). We used the *core* function from the phyloseq package v1.38.0 to determine common bacterial, with a minimum abundance threshold of 0.001% and minimum prevalence of 90% across three levels: (1) all samples, (2) only king penguins, and (3) only Magellanic penguins.

To determine differences in alpha diversity across intraspecific and interspecific comparisons, we used the Shannon index and Faith’s Phylogenetic Diversity (PD, hereafter), with phyloseq v1.38.0 and picante v1.8.2 packages, respectively (McMurdie & Holmes, 2013; Kembel et al., 2010). To determine intraspecific and interspecific differences across body sites, we compared alpha diversity with the Kruskal-Wallis test. Significant results were further addressed to detect specific differences with the *post hoc* Wilcoxon ranks sum test, with Holm *p*-value adjustment for multiple comparisons.

To determine intraspecific and interspecific differences in bacterial community structure (*i.e.,* beta diversity) between penguin body sites, we used weighted unifrac distances (Lozupone et al., 2011). We compared beta diversity with permutational analysis of variance (PERMANOVA) (Anderson & Walsh, 2013). Upon significant results, *post hoc* paired PERMANOVA tests with *p*-value Holm adjustment for multiple comparisons were performed to detect specific differences between body sites. We compared beta diversity dispersion between sample types with the permutest function using the vegan package v2.6.4 (Oksanen et al., 2022). Dispersion analysis was needed to judge the adequacy of PERMANOVA results, since the PERMANOVA test may confound location and dispersion effects when there are unbalanced designs or differences between dispersion among tested factors (Anderson & Walsh, 2013). We explored ASVs responsible for group differentiation with the simper (similarity percentage) analysis (Clarke, 1993), which includes ASVs that contributed to at least 70% of the differences among groups of interest.

To test the relevance of neutral processes in the assembly of penguin body site microbiota, we used the Sloan Neutral Community Model for prokaryotes (Sloan et al., 2006). We used the code provided by Burns et al. (2016) to implement this model in R. The Sloan neutral model was fitted to the observed frequency of occurrence of ASVs (*i.e.,* the proportion of body sites in which each ASV is detected) and their abundance in the metacommunity (*i.e.,* estimated by the mean relative abundance across all body sites) by a parameter describing the migration rate *m*. The estimated migration rate is the probability that a random loss of an individual in a local community will be replaced by dispersal from the metacommunity, thus it can be interpreted as a measure of dispersal limitation. The general prediction is that abundant taxa in the metacommunity (*i.e.,* all penguin body sites from each species) will have higher frequency across local communities (*i.e.,* specific penguin body sites) since they are more likely to disperse by chance and be randomly sampled. In contrast, rare taxa are more likely to be lost from individual communities (individual body sites) due to ecological drift. Communities whose microbial taxa distribution adjusts to this prediction will have higher *R*^2^ values. The fit of the neutral model is compared with the fit of a binomial distribution model to determine whether incorporation drift and dispersal limitation (*m* parameter) improve the fit of a model beyond random sampling from the source metacommunity (Sloan et al., 2007). A comparison between the fit of the Neutral and Binomial models was conducted with the Akaike information criterion (AIC) of each model calculated in R. In addition, we explored the bacterial genera that fell above and below predicted frequency categories and plotted the five bacterial genera with more ASVs.

## Results

### DNA yield does not differ among penguin sample types but it does between penguin swabs and nest soil swabs

DNA yield differed subtly between king penguin sample types (KW = 6.823, *p* = 0.032). However, when we performed the multiple paired comparisons Wilcoxon test, these differences did not hold. DNA yield from chest feathers did not differ from back feather swabs (alpha = 0.05). Comparisons with the greatest variability in its DNA yield were back swab *vs* chest feather (p.adjusted = 0.086) and chest feather *vs* foot swab (p.adjusted = 0.086). While swabs (back *vs* foot) had similar DNA yields (p.adjusted = 0.611).

In contrast, in Magellanic penguins, DNA yield differed across sample types (KW = 15.323, *p* = 0.001). The Wilcoxon multiple paired comparisons test revealed that the main differences were driven by nest swabs *vs* body site sample types, specifically nest swab *vs* back swab (p.adjusted = 0.011) and nest swab *vs* foot swab (p.adjusted = 0.011). However, chest feathers’ DNA yield did not differ from the nest soil’s (p.adjusted = 0.118). Despite differences in sample types (*i.e.,* penguin feather swabs *vs* penguin chest feathers) penguin body site samples had similar DNA yields; back swab *vs* chest feather (p.adjusted = 0.254), back swab *vs* foot swab (p.adjusted = 0.798), chest feather *vs* foot swab (p.adjusted = 0.254).

### Amplicon sequence variant performance and taxonomic characteristics of penguin sample types

All the samples (*n* = 64) gave useful data after strict quality filters (Table 1). Decontam pipeline flagged 117 ASVs distributed in 6,672 sequences as possible contaminants. Before conducting statistical analyses, we excluded these sequences. In total, after sample rarefying, we obtained 1,926,400 bacterial sequences distributed in 17,005 amplicon sequence variants (ASVs) across all samples (from body sites and nest soil of Magellanic penguins).

**Figure 2:**
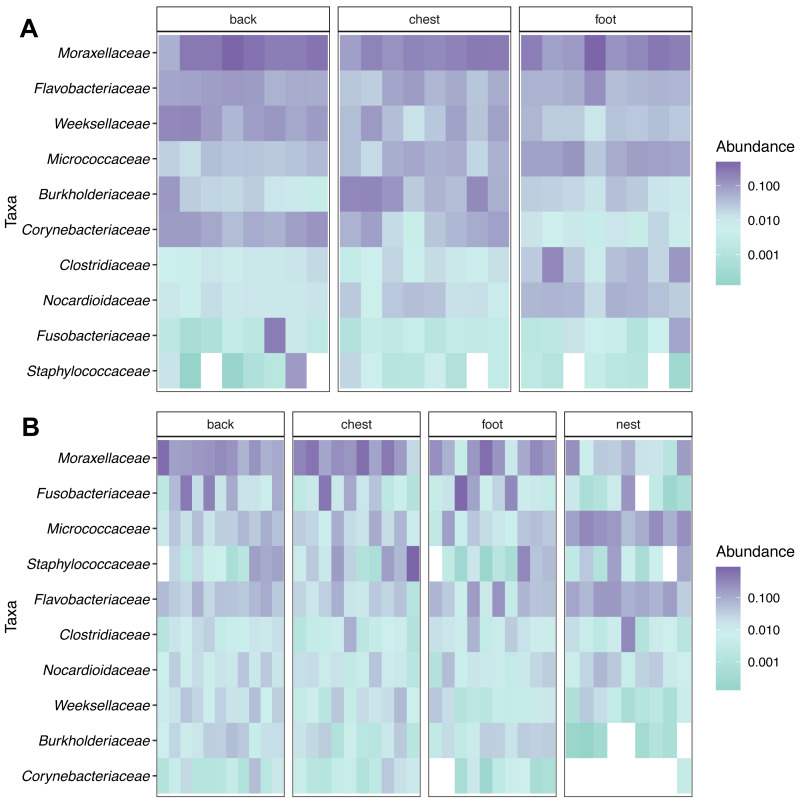
Relative abundance heatmap of the 10 most abundant bacterial families across penguin samples. (A) King penguin body site samples. (B) Magellan penguin body site and nest soil samples.

Taxonomic composition analyses showed that *Moraxellaceae* was the most abundant family across body sites in both king (Fig. 2A) and Magellanic penguins (Fig. 2B). In contrast, Magellanic penguin nests were dominated by *Micrococcaceae* bacteria (Fig. 2B). The three most abundant families across king penguin body sites were: *Moraxellaceae* (27%), *Weeksellaceae* (11%), and *Flavobacteriaceae* (7%) on the back; *Moraxellaceae* (18%), *Burkholderiaceae* (10%), and *Micrococcaceae* (5%) on the chest; *Moraxellaceae* (21%), *Micrococcaceae* (7%), and *Flavobacteriaceae* (6%) on the foot. In Magellanic penguin, the three most abundant families across samples were: *Moraxellaceae*, (21%), *Fusobacteriaceae* (11%), and *Flavobacteriaceae* (5%) on the back; *Moraxellaceae* (29%), *Staphylococcaceae* (13%), and *Fusobacteriaceae* (7%) on the chest; *Moraxellaceae* (19%), *Flavobacteriaceae* (6%), and *Staphylococcaceae* (4%) on the foot; *Micrococcaceae* (17%), *Flavobacteriaceae* (11%), and *Moraxellaceae* (6%) on the nest.

The most abundant genus across penguin body sites in both species was *Psychrobacter*, particularly in the king penguin where it was the dominant taxa in almost all body sites (Table S1). However, Magellanic penguin samples showed heterogeneity of dominant taxa. Specifically in nest soil samples, where most samples (5/9) were dominated by an unknown genus of the *Micrococcaceae* family, while each of the other nest samples was dominated by a different genus: *Arthrobacter* (*Micrococcaceae*), *Clostridium sensu stricto 1* (*Clostridiaceae*), *Psychrobacter*, and *Vibrio* (*Vibrionaceae*) (Table S1).

Phylogenetic exploration of *Psychrobacter*-related sequences distribution in penguins’ body sites revealed that these sequences were widespread across penguin body sites. In the king penguin, most *Psychrobacter*-related sequences were widespread among body sites, whereas in Magellanic penguins there were some *Psychrobacter* sequences specific to body sites, while a subset of them co-occurred in nest soil (Fig. S3).

### Most ASVs are unique to each penguin body site, yet there is a core among all samples

**Figure 3:**
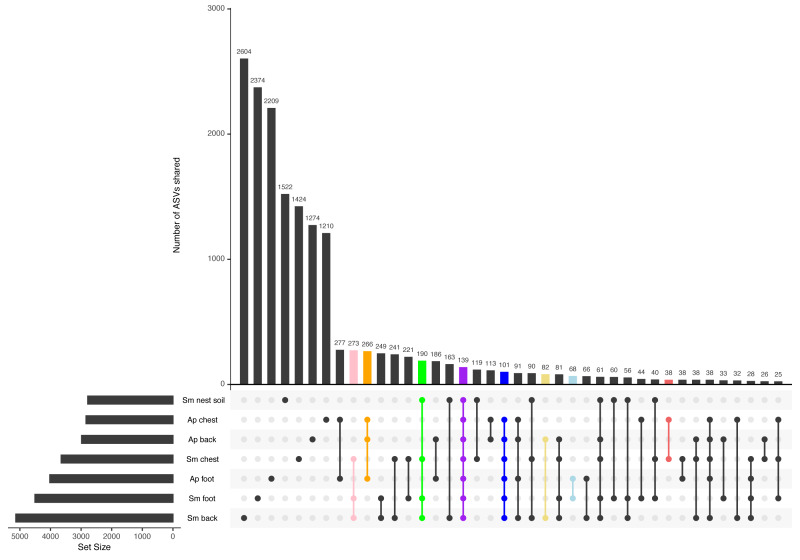
Upset plot between all penguin sample types. The pink bar highlights ASVs shared by Magellanic penguin (Sm) body sites (273); the orange bar highlights ASVs shared by king penguin (Ap) body sites (266); the green bar highlights ASVs shared by Magellanic penguin samples (190); the purple bar highlights ASVs shared by all penguin samples (139); the blue bar highlights ASVs shared by all penguin body site samples (101); the yellow bar highlights ASVs shared by penguins’ backs (82); the blue bar highlights ASVs shared by penguins’ feet (68); and the red bar highlights ASVs shared by penguins’ chests (38).

Most ASVs detected in this study were specific to each body site (Fig. 3). The site with the most unique ASVs was the Magellanic penguin back (2,604 ASVs), followed by Magellanic penguin foot (2,374), and king penguin foot (2,209) (Fig. 3). Intraspecific shared ASVs were few: 266 ASVs were shared among king penguin body sites (Fig. 3, orange bar), while 273 ASVs (Fig. 3, pink bar) were shared among Magellanic penguin body sites, and 190 among all Magellanic penguin samples (*i.e.,* body sites and nest soil) (Fig. 3, green bar). Interestingly, there were interspecific cores at different levels. Considering all penguin body sites, 101 ASVs were shared (Fig. 3, blue bar), while 139 ASVs were shared among all sample types (Fig. 3, purple bar). Considering core bacteria among paired body sites comparisons, 82 ASVs were shared among penguins’ backs (Fig. 3, yellow bar), 68 among penguins’ feet (Fig. 3, light-blue bar), and 38 among penguins’ chests (Fig. 3, red bar).

When considering the abundance threshold in core bacteria, we found different patterns. We explored core bacteria with a minimum relative abundance of 0.001% present in 90% of all sample types at interspecific and intraspecific levels. Interspecific core bacteria were: ASV8_*Clostridium sensu stricto* 1 and ASV10_*Micrococcaceae*. Magellanic penguin samples did not have additional core bacteria. In contrast, in king penguins, the bacterial core had more diversity in its composition and consisted of 16 taxa. Some taxa had several ASVs, such as *Psychrobacter* (7 ASVs) and *Corynebacterium* (2 ASVs). The rest of the core was completed by single ASVs of different genera: ASV2_*Ralstonia*, ASV8_*Clostridium sensu stricto 1*, ASV28_*Sporosarcina*, ASV26_*Ornithobacterium*, ASV24_*Tomittella*, ASV17_*Sphingomonas*, and ASV16_*Paeniglutamicibacter*.

### Microbiota alpha diversity has subtle differences across penguin samples

**Figure 4:**
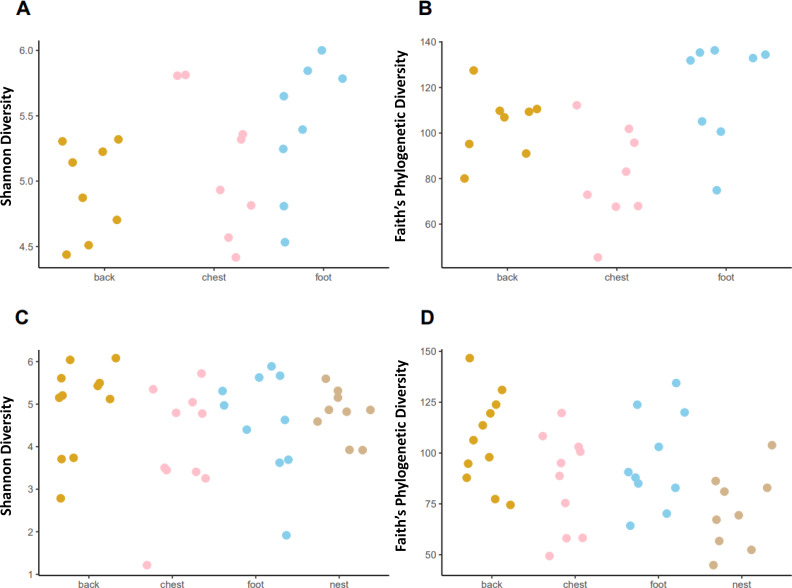
Alpha diversity measures, Shannon index and Faith’s Phylogenetic Diversity (PD) across penguin sample types. Alpha diversity measures across king penguin body sites, (A) Shannon and (B) *PD* diversity. Alpha diversity measures across Magellanic penguin body sites and nest soil, (C) Shannon and (D) *PD* diversity.

Faith’s phylogenetic diversity (*PD*) followed different patterns in each penguin species. In the king penguin, *PD* diversity was different across body sites (Fig. 4, KW = 8.465, *p* = 0.014). *Post hoc* paired comparisons revealed that the main difference was between chest *vs* foot (p. adj = 0.03), while chest *vs* back (p. adj = 0.13) and back *vs* foot (p. adj = 0.16) were similar. There were no nest samples in this species, as they do not dig burrows to nest, rather they nest on open land. In Magellanic penguin, *PD* diversity differed across sample types (Fig. 4, KW = 9.958, *p* = 0.018). *Post hoc* paired comparisons revealed that the main difference was between back *vs* nest (p.adj = 0.023), while chest *vs* nest (p.adj = 0.534) and foot *vs* nest (p.adj = 0.152) were similar. In contrast with king penguins, Magellanic penguin *PD* diversity was similar throughout body sites; back *vs* chest (p. adj = 0.452); back *vs* foot (p. adj = 0.684); and chest *vs* foot (p. adj = 0.684). Similarly, Shannon diversity was similar across both king penguin body sites (Fig. 4, KW = 3.785, *p* = 0.15) and Magellanic penguin samples (Fig. 4, KW = 4.09, *p* = 0.251). Overall, interspecific penguin body site alpha diversity comparisons were similar, with greatest differences in *PD* across penguins’ feet (Fig. S4; KW = 3.821, *p* = 0.05), yet it was similar between penguins’ backs (Fig. S4; KW = 0.027, *p* = 0.868) and penguins’ chests (Fig. S4; KW = 0.197, *p* = 0.656). Similarly, Shannon diversity was similar across penguins’ feet (Fig. S4; KW = 2.557, *p* = 0.109) and penguins’ backs (Fig. S4; KW = 0.681, *p* = 0.409), while penguins’ chests tended to differ (KW = 3.481, *p* = 0.062).

### Penguins’ body site microbiota structure follows species-specific patterns

**Figure 5:**
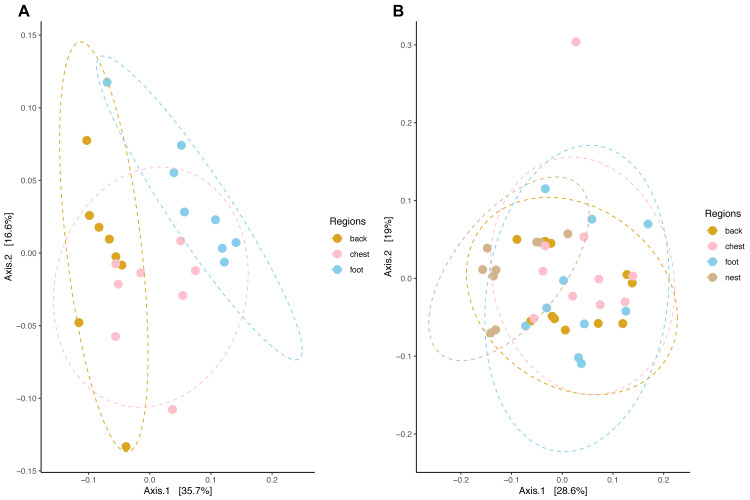
Principal coordinate analysis applied on weighted unifrac distances across penguin samples. (A) King penguin body site ordination. (B) Magellanic penguin body site and nest soil ordination.

Community composition structure measured by weighted unifrac distance displayed species-specific patterns. In king penguins, microbiota clustered accordingly to its body site, which in turn differed in their composition (PERMANOVA, pseudo-F = 4.693, *R*^2^ = 0.308, *p* = 0.001) (Fig. 5). Posterior PERMANOVA paired comparisons revealed that all king penguin body site microbiotas differed in their composition (Paired PERMANOVA results; back *vs* foot, pseudo-F = 7.008, *R*^2^ = 0.333, p. adj = 0.001, foot *vs* chest, pseudo-F = 3.505, *R*^2^ = 0.200; p. adj = 0.006; back *vs* chest, pseudo-F = 3.862, *R*^2^ = 0.216, p. adj = 0.001). Simper analysis indicated that ASVs within the bacterial genera *Psychrobacter*, *Corynebacterium*, *Clostridium sensu stricto 1*, *Ralstonia,* and *Fusobacterium* had the highest influence in the compositional differences between king penguin body sites (Table S2).

In king penguins, microbiota structure across body sites was not influenced by dispersion differences among body site microbiota (permutest *F* = 0.034, *p* = 0.971), hence body site microbiota structure was a consequence of centroid (*i.e.,* compositional) differences. In contrast, in Magellanic penguin microbiota weighted unifrac distance did not retrieve any distinctive cluster (Fig. 5), yet bacterial composition differed among sample types (PERMANOVA, pseudo-F = 2.701, *R*^2^ = 0.183, *p* = 0.001). Posterior PERMANOVA paired comparisons revealed that Magellanic penguin microbiota consistently differed between body sites and nest soil, while body site microbiota had similar composition (Table S3). Simper analysis indicated that ASVs within the bacterial genera *Psychrobacter* and *Fusobacterium*, as well as within the families *Staphylococcaceae* and *Micrococcaceae* had the highest influence in the compositional differences between Magellanic penguin body sites and nest soil microbiota (Table S2). As occurred in king penguin samples, Magellanic penguin body site, and nest soil microbiota had similar dispersion (permutest *F* = 1.403, *p* = 0.262), hence results were not confounded by dispersion heterogeneity among body site samples.

**Figure 6:**
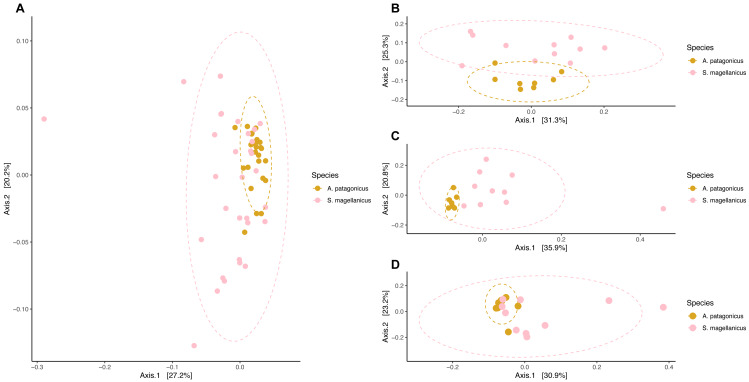
Principal coordinate analysis applied on weighted unifrac distances to conduct penguin body site interspecfic comparisons. (A) Interspecific comparison between all penguin body sites considered. (B) Penguins’ back comparison. (C) Penguins’ chest comparison. (D) Penguins’ foot comparison.

We detected compositional differences among species body site microbiota (pseudo-F = 4.556, *R*^2^ = 0.079, *p* = 0.001) (Fig. 6). Paired interspecific body site comparisons revealed differences between penguins’ backs (pseudo-F = 3.845, *R*^2^ = 0.184, *p* = 0.002); penguins’ chests (pseudo-F =3.670, *R*^2^ = 0.186, *p* = 0.002); and penguins’ feet (pseudo-F =2.254, *R*^2^ = 0.123, *p* = 0.014). However, it remained uncertain if these effects were caused by differences in centroid location or by dispersion heterogeneity since dispersion differed among penguin species (permutest *F* = 11.665, *p* = 0.001) and penguins’ body sites (backs, permutest *F* = 10.142, *p* = 0.009; chests, permutest *F* = 7.671, *p* = 0.001; feet, permutest *F* = 14.87, *p* = 0.001).

### Neutral processes influence body site microbiota assembly in penguins

**Figure 7:**
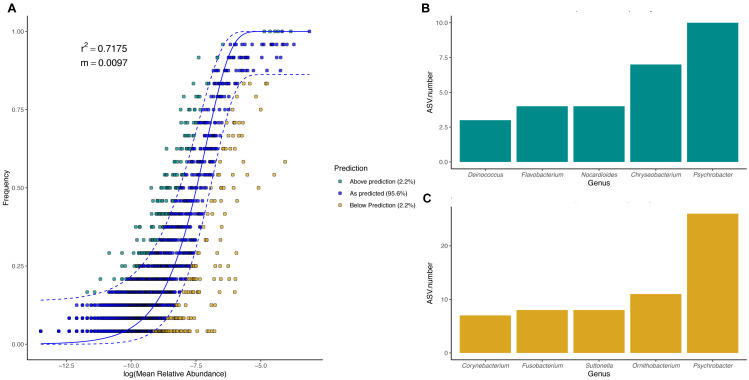
King penguin body site microbial taxa fit to Sloan Neutral Community Model. (A) Sloan neutral community model applied to king penguin body site microbiotas. (B) Bacterial genera with most ASVs whose frequency is above predicted frequency by the neutral model. (C) Bacterial genera with most ASVs whose frequency is below predicted frequency by the neutral model.

**Figure 8:**
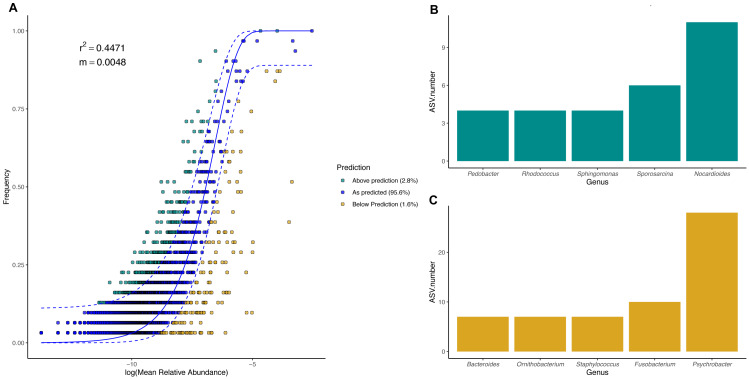
Magellanic penguin body site microbial taxa fit to Sloan neutral community model. (A) Sloan neutral community model applied to Magellanic penguin body site microbiotas. (B) Bacterial genera with most ASVs whose frequency is above predicted frequency by the neutral model. (C) Bacterial genera with most ASVs whose frequency is below predicted frequency by the neutral model.

To evaluate the relevance of the neutral processes in penguin body site microbiota assembly, we fitted a neutral model with all penguin body sites from each species. Overall, microbial taxa distribution in penguin body sites was well described by neutral models in both king (Fig. 7) and Magellanic penguins (Fig. 8). Nevertheless, the relevance of the neutral processes in body microbiota assembly was higher in the king (*R*^2^ = 0.7175) than in Magellanic penguin (*R*^2^ = 0.4471). Yet, in both species, neutral models that incorporate ecological drift and dispersal limitation (AIC score in the king 870.747 and Magellanic penguin 1819.09) were more informative than the binomial model alone (Binomial scores in the king 884.56 and Magellanic penguin 1833.48). Estimated migration rates were low in both species (Magellanic penguin, *m* = 0.0048; king penguins, *m* = 0.0097), indicating the low probability that microbes from the metacommunity could fill vacancies left in local communities.

In general, most ASVs abundance and frequency detection fit the neutral model predictions in both species, yet several ASVs did not fit this neutral prediction. In the king penguin, there were 162 ASVs whose frequency detection was above the model prediction, while 161 ASVs’ frequency detection was below the model prediction (File S1). *Psychrobacter* was the genus with the most ASVs whose frequency detection was above and below prediction (10 ASVs and 26 ASVs, respectively) (Fig. 7). In the Magellanic penguin there were 279 ASVs whose frequency detection was above model prediction, while 158 ASVs frequency detection was below model prediction (File S2). *Nocardioides* was the genus with more ASVs whose frequency detection was above model prediction (11 ASVs), whereas *Psychrobacter* was the genus with more ASVs whose frequency was below model prediction (28 ASVs) (Fig. 8).

## Discussion

We investigated the effect of body site on microbiota diversity and composition in two penguin species inhabiting the Strait of Magellan, the Magellanic and the king penguin. To the best of our knowledge, this is the first report addressing body site microbiota in penguins. Our main findings highlighted the prevalence of *Moraxellaceae* bacteria in all penguin body sites, particularly *Psychrobacter*. We found partial support for our first prediction, which stated that microbiota diversity and composition vary across body sites due to differential environmental exposure. We found that penguin body site microbiota alpha diversity tended to vary in the king penguin, whereas it remained similar across Magellanic penguin body sites. Similarly, we detected compositional differences throughout king penguins’ body site microbiota, whereas body site microbiota composition remained similar in Magellanic penguins. Interestingly, we detected compositional differences between all Magellanic penguin body sites and nest soil microbiota. We found partial support for our second prediction, which stated that microbiota diversity and composition vary between similar penguin body sites (*e.g.*, king back *vs* Magellanic back). Interspecific alpha diversity comparisons tended to differ between penguins’ feet (phylogenetic diversity) and penguins’ chests (Shannon diversity). We detected interspecific compositional differences between penguin body site microbiota. Nevertheless, our evidence requires caution, since estimated dispersion was different between Magellanic and king penguin body site microbiota, hence it was uncertain to which degree microbiota composition differed between species. Finally, we found that body site microbiota was influenced by neutral processes in both species, particularly in the king penguin (king penguin, *R*^2^ = 0.7112, Magellanic penguin *R*^2^ = 0.4475).

### Penguin microbiota alpha diversity was similar across body sites

We predicted differences in microbiota alpha diversity among each penguin species body site, yet we found low support for this prediction. Phylogenetic diversity was the only metric that retrieved significant (but subtle) results, but only between the chest and foot microbiota of the king penguin, whereas in the Magellanic penguin, microbiota alpha diversity remained similar across body sites.

Our results are in contrast with evidence from continental avian species, which reported differences in avian body site microbial alpha diversity (Van Veelen, Falcao Salles & Tieleman, 2017; Engel et al., 2020). However, our results are in line with those that showed that seabird body sites have similar microbial alpha diversity (Leclaire et al., 2019; Leclaire et al., 2023). Penguin body site microbiota alpha diversity similarity might point to environmental similarities in each body site, that render similar filters to microbial colonization, despite the inherent differences between plumage and feet skin. This might be particularly true for the Magellanic penguin, whose body site microbiota alpha diversity had stronger similarities. This could be explained by the fact that Magellanic penguins rest and nest in burrows, where they are protected from environmental stressors that could influence body site microbiota diversity and composition, like solar radiation or wind (Saranathan & Burtt, 2007; Graves et al., 2020). Underground sheltering might provide similar abiotic conditions, resulting in similar microbial diversity across penguin body sites.

We also predicted differences in body site microbiota alpha diversity between penguin species. However, we found poor support for this hypothesis, since interspecific body site microbiota alpha diversity only differed weakly between the phylogenetic diversity of penguins’ feet and tended to differ between Shannon diversity of penguins’ chests. This could be explained by a lack of selective pressure on penguin body sites for bacterial growth, or little bacterial control mechanisms. For example, preen oil is a substance produced in the preen gland that birds apply to their feathers during preening. The main roles of preen oil in continental birds are for the maintenance of feather quality and restriction of bacterial proliferation (Shawkey, Pillai & Hill, 2003). In those birds, it has been observed that preen oil chemical composition changes seasonally, hence bacterial control in feathers fluctuates over the year (Reneerkens, Piersma & Sinninghe Damsté, 2002; Reneerkens et al., 2008). Alternatively, preen oil in penguins from cold environments could be related to different roles, such as hydrophobicity and ice formation delay in its plumage (Alizadeh-Birjandi et al., 2020).

Given that penguins fast during austral spring to rear their egg, we could speculate that their preen oil might have fewer antibiotic properties, due to a potential tradeoff between nutrient allocation for penguin energetic metabolism and hygienic mechanisms caused by fasting. Consequently, penguin body sites in austral spring could have equivalent filters to microbial colonization that result in body site microbiota with similar alpha diversity, despite differences in breeding ecological strategies. Future studies that simultaneously address preen oil chemical composition, bacterial load regulation, and body site microbiota diversity across their breeding phenology, will be useful to test this idea. In addition, an important limitation of our study relies on missing important covariables that influence seabird body site microbiota alpha diversity, like fitness status (Leclaire et al., 2023), major histocompatibility allele diversity (Leclaire et al., 2019) or sex-specific factors (Pearce et al., 2017). Testing these covariables might be an interesting topic for future research in penguin–bacteria interactions. In future studies we will link feather microbiome with transcriptomics and host genetics as well as isotopic signatures, probably allowing us to test such variables.

### Body site microbiota structure across intraspecific and interspecific patterns

We predicted differences in body site microbiota composition between each penguin species. We found partial support for this hypothesis. On one hand, the microbiota composition of king penguins differed across their body sites. The bacterial genera with the most influential ASVs responsible for this structure were *Psychrobacter* and *Corynebacterium*. On the other hand, the composition of the Magellanic penguin body site microbiota was similar throughout its body. In birds, the effect of body site on microbiota composition is driven by ecological factors. Continental species, which are in constant contact with soil tend to have similar microbiota composition throughout its body and with the environment (Van Veelen, Falcao Salles & Tieleman, 2017; Engel et al., 2018). On the contrary, in seabirds, microbiota composition differs throughout body sites (Pearce et al., 2017; Leclaire et al., 2019; Leclaire et al., 2023). When framing our results with avian body site microbiota evidence, king penguins adjust to the seabird’s pattern, where microbiota composition differs throughout its body sites. Conversely, Magellanic penguins fit the pattern of continental birds, where body site microbial composition is similar across their body.

Body site microbiota is expected to change throughout the body whenever differential environmental exposure and body site-specific abiotic conditions are met (Grice et al., 2009; Costello et al., 2009). Contrasting ecological strategies among our studied species might influence the observed patterns. On one hand, king penguins that nest on open land might experience stronger environmental exposure than Magellanic penguins, which shelter underground in their burrow. In king penguins, differential environmental exposure might influence abiotic conditions of each body site (*e.g.*, the back is more exposed to wind and solar radiation, whereas the feet have less exposure), resulting in microbiota compositional differences throughout the body. On the other hand, Magellanic penguins sheltering underground might experience similar abiotic conditions that render similar microbial compositional profiles across their body sites. A possible additional selective pressure influencing penguin body site microbiota is their unique subaquatic lifestyle (De Roy, Jones & Cornthwaite, 2022). Penguins are specialized in diving, yet diving depth differs across species. For instance, Magellanic penguin dive depth differs among colonies, but dives can be as deep as 90 m (Walker & Boersma, 2003). In contrast, king penguins can dive very deep, up to 440 m (Charrassin, Maho & Bost, 2002). Diving and its associated changes in pressure and temperature add another abiotic filter to penguin body site microbiota assembly, that appears unique among birds.

Furthermore, we predicted interspecific differences among paired body site microbiota compositional comparisons. We found compositional differences between all paired body site comparisons (*e.g.*, king back *vs* Magellanic back), hence we found support for our prediction. However, this is a cautionary interpretation, since dispersions among penguins’ body sites were not homogeneous. This issue is common when comparisons are conducted under unbalanced designs (*i.e.,* the Magellanic penguin sample size was higher) (Anderson & Walsh, 2013). Dispersion heterogeneity among conditions tested confounds community structure differentiation by niche centroid and dispersion differences, hence we could not conclude about interspecific penguin body site microbiota compositional differentiation. Yet, it is important to frame this issue under the specific ecological context of each species. For instance, Magellanic penguins in Contramaestre Island are in contact with sea lions and several bird species on the island and coast (*e.g.*, seagulls, brown skua, imperial cormorant), and inside their nest with rabbits and conspecifics. These dynamic interspecific interactions might enhance dispersion in microbial communities inhabiting Magellanic penguin body sites. Under this dynamic environment, contingent factors (*e.g.*, which bacteria arrived first at penguin body sites) might have more relevance in body site microbiota structure, and at the same time promote interindividual dispersion (Fukami, 2015).

### Nest microbiota had a poor influence on Magellanic penguin body site microbiota

We found differences between all Magellanic penguin body sites and nest soil microbiota. Influential ASVs responsible for this structure were *Psychrobacter*, *Fusobacterium*, and from *Staphylococcaceae* and *Microccocaceae* families. This is a surprising result, since evidence from continental birds indicates that plumage is heavily influenced by the environmental microbiome, such as carcasses in vultures (Roggenbuck et al., 2014), vegetation in arboreal birds (Dille, Rogers & Schneegurt, 2016), and soil microbes in birds that forage or nest in the ground (Burtt Jr & Ichida, 1999; Saag et al., 2011; Goodenough et al., 2017; Van Veelen, Falcao Salles & Tieleman, 2017, Van Veelen, Falcao Salles & Tieleman, 2018). This is particularly true for birds that nest in burrows, since burrow digging enhances horizontal transmission from soil microbes to avian body sites (Kilgas et al., 2012). Thus, whenever spatial environment and ecological traits are shared, convergence in avian body site microbiota composition is expected (niche environmental hypothesis, Van Veelen, Falcao Salles & Tieleman, 2017). The niche environmental hypothesis has been adequate in continental bird species, nevertheless, there are exceptions, such as redstarts (Bisson et al., 2007) and seabirds (Pearce et al., 2017). Our results found low support for the niche environmental hypothesis in the Magellanic penguin, hence its body site microbiota had low compositional similarity with nest soil microbiota. Magellanic penguin body sites were characterized by *Psychrobacter* (*Moraxellaceae*) taxa, whereas nest soil microbiota was characterized by *Microccoccaceae* taxa. Interestingly, nest microbiota also had *Psychrobacter*-related sequences. These sequences were likely a subset of Magellanic penguin body sites since *Psychrobacter* sequences related to nest soil were also detected in Magellanic penguin body sites. Whether these sequences were transmitted by the penguin to the soil, or the penguin acquired them from the soil is a “chicken/egg” conundrum that we cannot answer at this moment. However, it is a relevant topic to address in further studies to understand the role of nest microbiota in the Magellanic penguin body site microbiota assembly.

Interestingly, we found high heterogeneity in dominant taxa inhabiting Magellanic penguin nests. Five nests were dominated by an unknown genus from the *Micrococcaceae* family, while the other nests were dominated by different genera, such as *Arthrobacter*, *Clostridium sensu stricto 1*, *Psychrobacter*, and *Vibrio*. This heterogeneity in nest soil dominant bacteria might enhance dispersion in Magellanic penguin body sites. In addition, this could partially explain the lower prevalence that *Psychrobacter* has in Magellanic penguin body sites (back 4/11, chest 7/10, and foot 4/10). Given the high heterogeneity in the nest microbial profile, it could be plausible that each Magellanic penguin–nest combination creates idiosyncratic body site microbiota compositions across penguins. Comparative studies involving Magellanic penguin colonies with different environmental characteristics (*e.g.*, vegetation cover) might serve to test if differences in nest vegetation type are related to Magellanic penguin body site microbiota structure.

### Core bacteria across penguin sample types and species-specific core

Most bacteria were unique to each specific penguin body site, however, we detected a strong core (90% of prevalence threshold), although with a low relative abundance (0.001% minimum relative abundance) among all penguin samples. Common bacteria across penguin samples and nest soil were *Clostridium sensu stricto* and one *Micrococcaceae* taxa. Furthermore, when exploring species-specific core bacteria, we found a rich core of 15 taxa (using the same thresholds) in the king penguin. Core bacterial genera with more ASVs across king penguin body sites were *Psychrobacter* and *Corynebacterium*. *Psychrobacter* is a common member of birds and cold environments (detailed below) while *Corynebacterium* and *Clostridium* are common members of the gut and fecal microbiome of birds (Roggenbuck et al., 2014; Hird et al., 2015; Tian et al., 2021; Bodawatta et al., 2022; Grond, Louyakis & Hird, 2023). Although this core was detected with a low relative abundance (>0.1%), its consistency and prevalence might point to the constant contact that penguin body sites have with feces and environmental sources. These sources might differ between species since each species engage in unique interspecific interactions. For instance, king penguins at Pingüino Rey Natural Reserve interact with the upland goose (*Chloephaga picta*) and South American grey fox (*Lycalopex griseus*). On the contrary, Magellanic penguins in Contramaestre Island may interact with several other vertebrates, which might hinder the establishment of a diverse core across individuals, since various sources can influence body site microbial compositions.

### *Psychrobacter* is a common member of vertebrates’ surface microbiota

*Psychrobacter* is a common environmental bacterium in cold environments, such as sea ice, seawater, marine sediment, glaciers, ornithogenic soils, and bioaerosols (Bowman, 2006; Bowers et al., 2009; Chen et al., 2021). Besides its environmental occurrence, *Psychrobacter* is also associated with marine animal hosts, such as the skin and blow of humpback whales (Apprill et al., 2014; Apprill et al., 2017; Bierlich et al., 2018; Toro et al., 2021) and seal feces (Yassin & Busse, 2009; Banks, Craig Cary & Hogg, 2014). In addition, *Psychrobacter* has been detected in several avian species, such as white storks and gentoo penguin throat (Kämpfer et al., 2015; Kämpfer et al., 2020), seabirds ventral feathers and cloaca (Leclaire et al., 2019; Leclaire et al., 2023), finches feathers (Engel et al., 2018; Engel et al., 2020), bird eggs surface (Song et al., 2023) and even in Adélie penguin stomach content (Yew et al., 2017). Interestingly, *Psychrobacter* has been recorded in humpback whale skin from the Strait of Magellan (Toro et al., 2021) and Antarctic regions (Bierlich et al., 2018). Thus, it is likely that vertebrate hosts in the Strait of Magellan are exposed to a rich *Psychrobacter* environmental pool coming from local (*e.g.*, local glaciers) and regional sources (*i.e.,* Antarctic, and marine bioaerosols). Since *Psychrobacter* sequences represent the dominant genus in all body sites in both species, yet there are microdiversity patterns at each body site (*i.e., Psychrobacter* unique sequences), it is possible that different environmental *Psychrobacter* pools simultaneously colonize penguin body sites. Future studies must address the functional basis of this association.

### Species-specific importance of neutral processes in penguin body site microbiota assembly

We detected a good fit in microbial taxa abundance to a neutral community model in penguin body site microbiota. The model incorporating the migration parameter had a better fit, which indicates that ecological drift and dispersal limitation are relevant processes in penguin body site microbiota assembly. The Sloan neutral community model has become a valuable tool to explain the high compositional variability inherent to host-associated microbial communities (Burns et al., 2016; Venkataraman et al., 2015; Chiarello et al., 2019; Tong et al., 2019). Community assembly driven by neutral processes is expected to have high variability within local conditions (*e.g.*, body sites), which should create high dispersion among conditions, hindering clear clustering patterns as a function of the predictor variables (Costello et al., 2012; Sieber et al., 2019). Neutral processes might be an important factor influencing penguin body site microbiota assembly, since penguin body sites were highly variable, especially in Magellanic penguins. Yet, the influence of neutral processes varied between species, as shown by differences in fitness to the neutral model (king *R*^2^ = 0.71, Magellan, *R*^2^ = 0.44). Our results suggest that king penguins’ body site microbiota assembly might be influenced by an interplay of potential microenvironmental differences (suggested by body site microbiota structure) and ecological drift across body sites. Conversely, Magellanic penguins’ whole body site microbiota assembly might be influenced by the environmental shifts associated with sheltering and open exposure, as well as ecological drift.

We detected several bacterial genera whose frequency detection was above the prediction by the neutral community model. In the king penguin, these genera were mainly *Psychrobacter*, *Chryseobacterium*, and *Nocardioides*; in the Magellanic penguin, these were *Nocardioides*, *Sporosarcina*, and *Sphingomonas*. Conversely, bacterial genera whose frequency was below the prediction under the neutral model were mainly *Psychrobacter*, *Ornithobacterium*, and *Suttonella* in the king penguin; and *Psychrobacter*, *Fusobacterium*, and *Staphylococcus* in the Magellanic penguin. Given that *Psychrobacter* is the most prevalent bacterial taxa in both categories in the king penguin, and only in below predicted frequency category in the Magellanic penguin, it is tempting to speculate that these patterns reflect different *Psychrobacter* thermal ecotypes (Welter et al., 2021). *Psychrobacter* sequences under the above category might reflect strains with wide thermic tolerance, while those under the below category might reflect psychrophilic strains. Overall, bacterial genera under the above category might be oligotrophic bacteria, capable of withstanding dynamic environmental fluctuations, while bacterial genera under the below category might be bacterial genera with narrow environmental tolerance (*e.g.*, psychrophilic). Nevertheless, functional microbiome approaches are needed to test these ideas.

### Potential biases in microbiota profiling as a consequence of different sampling approaches

Initially, our goal was to use feathers to sample penguins’ back and chest plumage. As we proceeded with the sampling we noted that feather extraction increased animal stress so we changed to using sterile swabs. This sampling shift could have introduced bias in the microbiota profiling. However, we do not consider this a major caveat, since DNA yield between back swabs and chest feathers did not differ. Moreover, several characteristics of the plumage microbiota remain constant despite potential biases due to sampling differences, such as taxonomic profile, alpha diversity, and compositional dispersion. Furthermore, we only detected compositional differences across microbiota body sites in one species (*i.e.,* king penguins). We interpret this as evidence that sampling discrepancies did not influence our ability to identify variations in composition, but rather that it was determined by the biology of the species. However, we acknowledge the value of consistency through sampling (*i.e.,* either using only swabs or tip feathers for plumage sampling) and advocated to keep sampling as uniform as possible.

## Conclusions

We studied body site microbiota (back, chest, and foot) in two penguin species, the Magellanic and the king penguin, in two locations in the Strait of Magellan. Our results highlight the prevalence of different ASV variants within the *Psychrobacter* genus in all penguin body sites. Penguins’ body site microbiota structure follows species-specific patterns. On one hand, the king penguin showed the seabird pattern where microbiota body site composition differs throughout its body. On the other hand, the Magellanic penguin showed partially the continental bird pattern since its body site microbiota is similar throughout its body, but it is not influenced by its environment (*i.e.,* nest soil microbiota). Furthermore, both penguin species’ body site microbiota are influenced by neutral processes, which highlight the relevance of stochastic processes in body site microbial compositional variation. Given that the Strait of Magellan has several Magellanic penguin colonies, future studies are needed to determine the geographic prevalence of *Moraxellaceae* bacteria with the Magellanic penguin body. Likewise, metagenomic and cultivation assays would be useful in disentangling the metabolic basis that allows these bacteria to prevail in penguin body sites.

## Supplemental Information

10.7717/peerj.16290/supp-1 Supplemental Information 1 Rarefaction curve of King penguin body sites Body sites are displayed by different colors: blue, chest; green, foot; and red, back.

10.7717/peerj.16290/supp-2 Supplemental Information 2 Rarefaction curve of Magellanic penguin body sites and nest soil Sample types are displayed by different colors: blue, foot; green, chest; purple, nest soil; and red, back.

10.7717/peerj.16290/supp-3 Supplemental Information 3 Phylogenetic relationships between *Psychrobacter* sequences in penguin samples (A) *Psychrobacter* sequences in king penguin body sites. (B) *Psychrobacter* sequences in Magellanic penguin samples. Colors refer to sample type, while number of circles refer to the number of samples where the sequence occurred.

10.7717/peerj.16290/supp-4 Supplemental Information 4 Interspecific comparison of alpha diversity measures across penguin body sites (A), PD alpha diversity body site interspecific comparison. (B) Shannon index body site interspecific comparison.

10.7717/peerj.16290/supp-5 Supplemental Information 5 Most abundant bacterial genera and proportion of samples where it dominates

10.7717/peerj.16290/supp-6 Supplemental Information 6 ASVs identified using SIMPER analyses that drive microbiota differences between body sites in king penguin and between body sites and nest soil in Magellanic penguin

10.7717/peerj.16290/supp-7 Supplemental Information 7 Paired Permanova comparisons between Magellanic penguin (*Spheniscus magellanicus*) body sites and nest soil microbiota composition

10.7717/peerj.16290/supp-8 Supplemental Information 8 King penguin Sloan Neutral Community Model results

10.7717/peerj.16290/supp-9 Supplemental Information 9 Magellanic penguin Sloan Neutral Community Model results

10.7717/peerj.16290/supp-10 Supplemental Information 10 Code for community analysis reported in this article This code is related to data processing and community analysis.

10.7717/peerj.16290/supp-11 Supplemental Information 11 DNA concentration data from penguin samples and nest soil

## Additional Information and Declarations

### Field Study Permissions

The following information was supplied relating to field study approvals (*i.e.*, approving body and any reference numbers): Field studies were approved by Subsecretaría de Pesca y Acuicultura (E2021-531 & E- 3315).

### DNA Deposition

The following information was supplied regarding the deposition of DNA sequences: The raw 16S sequences are available at GenBank: PRJNA972831.

### Data Availability

The following information was supplied regarding data availability: The code and raw data are available in the Supplemental Files.
