# Supplementary figures and images for: Body site microbiota of Magellanic and king penguins inhabiting the Strait of Magellan follow species-specific patterns
Source: PeerJ. 2023 Nov 2;11:e16290. doi: 10.7717/peerj.16290 (PMC10625763; doi:10.7717/peerj.16290)

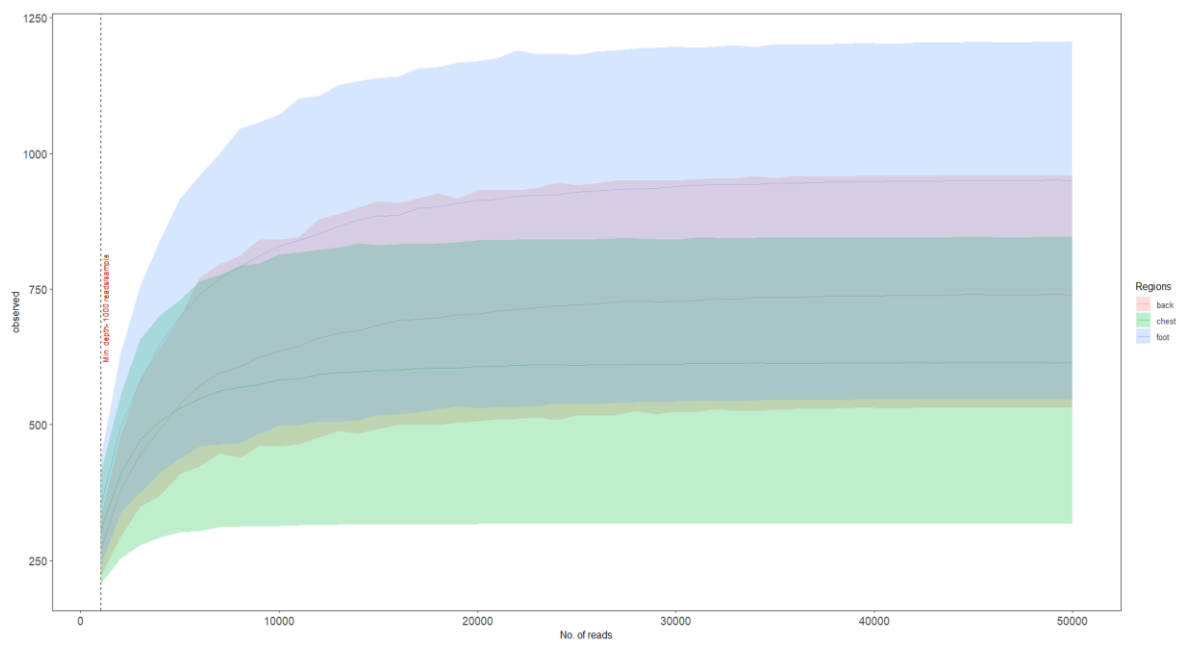

Supplement: Supplemental Information 1 — Body sites are displayed by different colors: blue, chest; green, foot; and red, back. [file peerj-11-16290-s001.pdf]

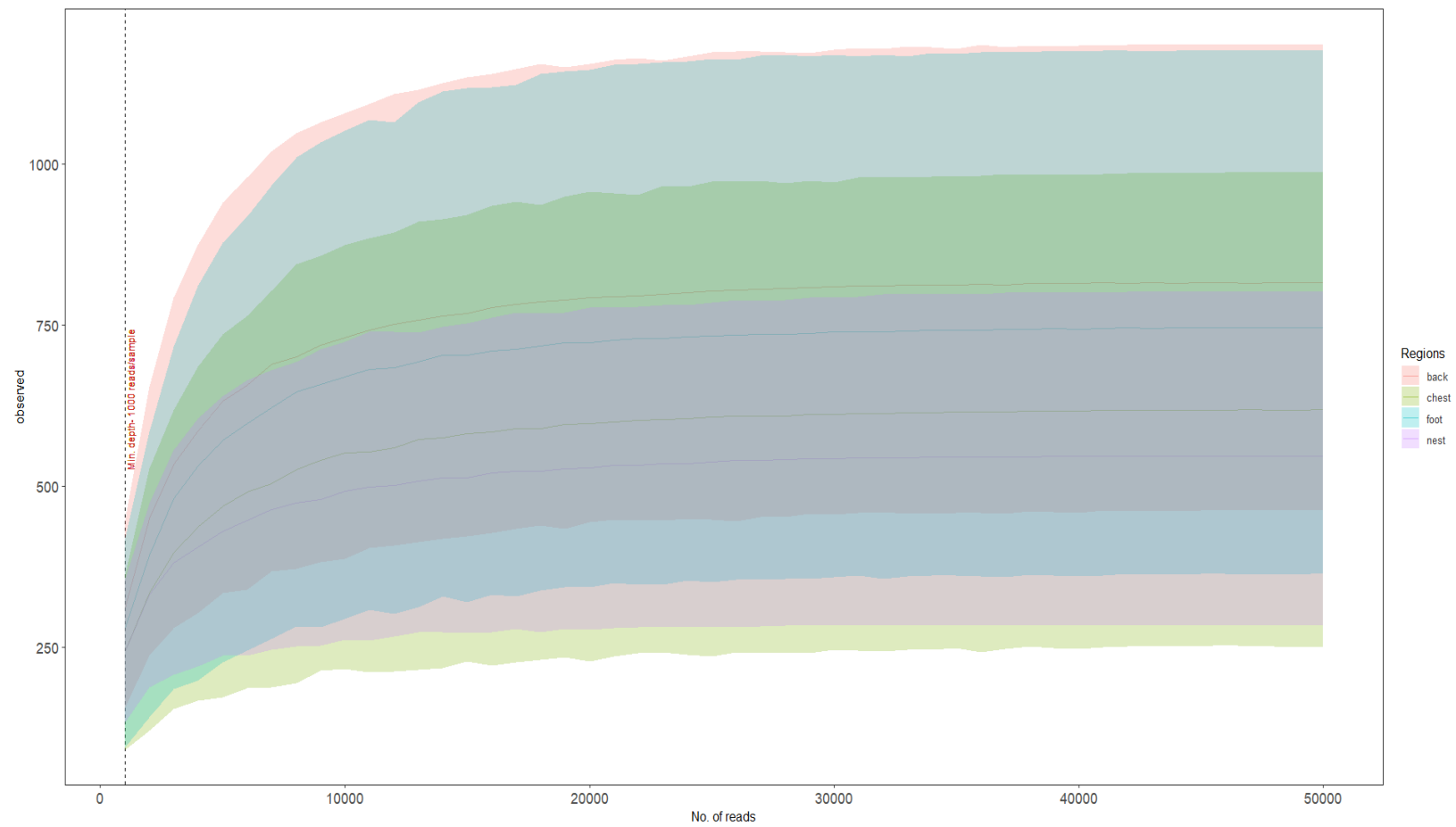

Supplement: Supplemental Information 2 — Sample types are displayed by different colors: blue, foot; green, chest; purple, nest soil; and red, back. [file peerj-11-16290-s002.pdf]

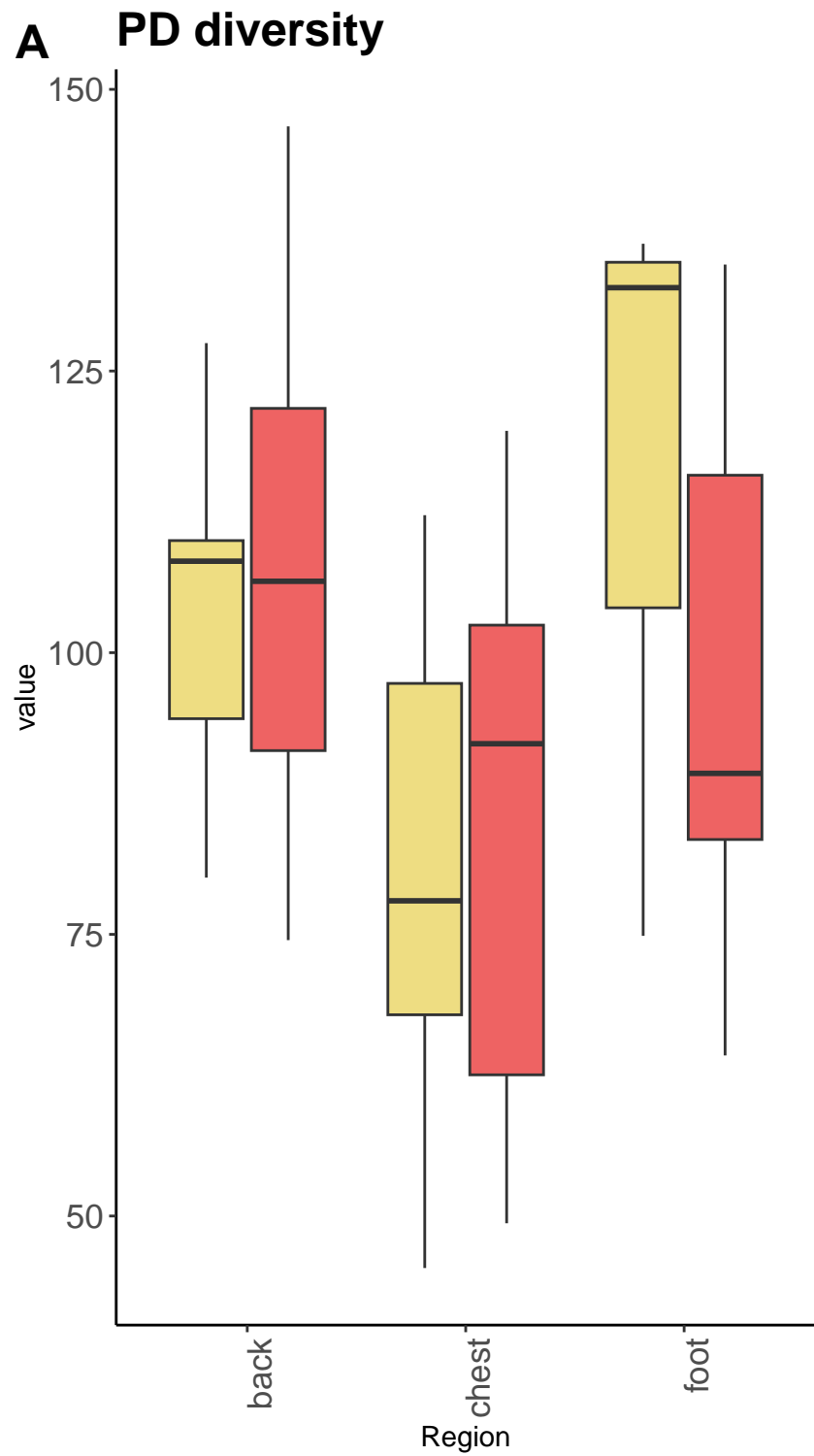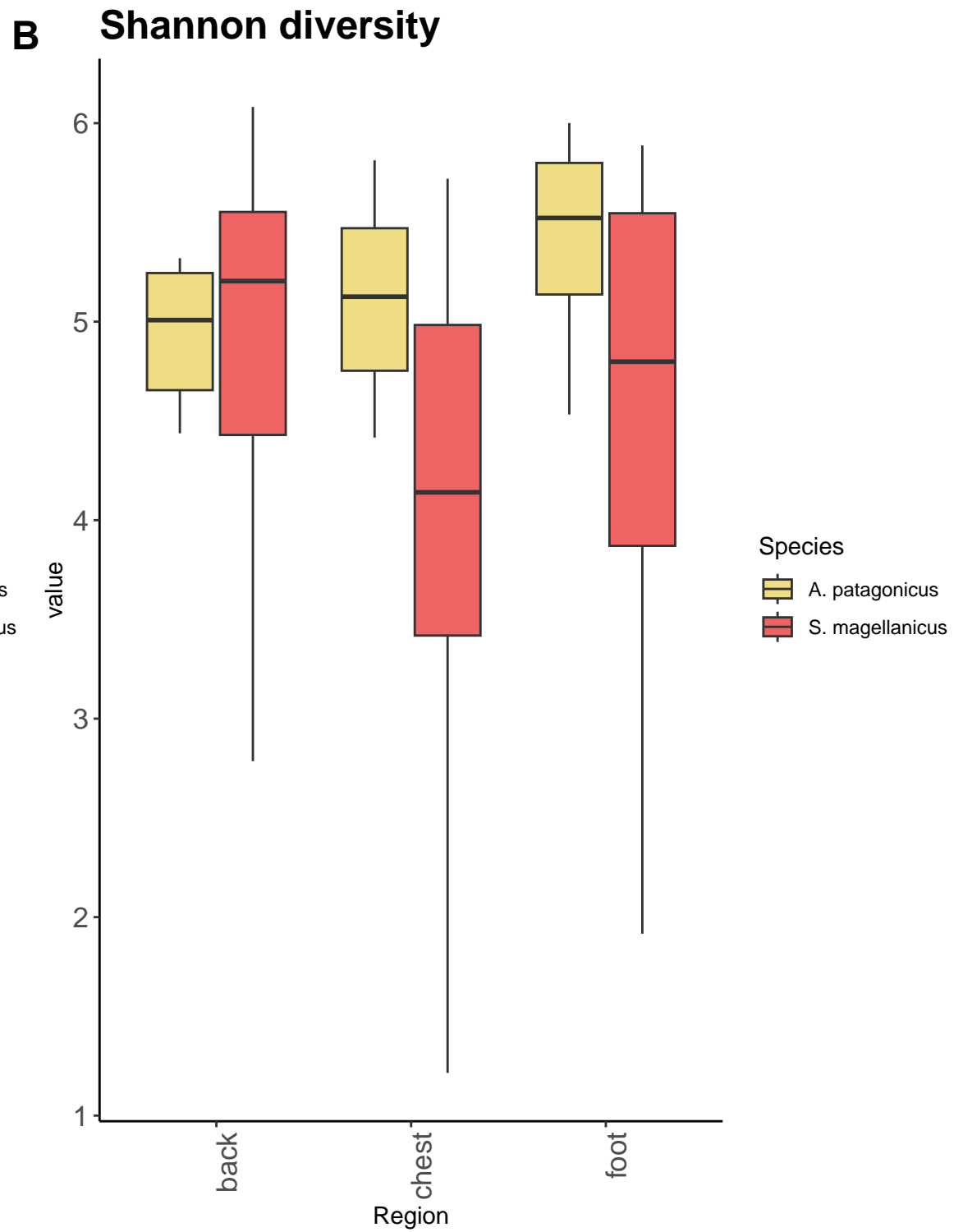

Supplement: Supplemental Information 4 — (A), PD alpha diversity body site interspecific comparison. (B) Shannon index body site interspecific comparison. [file peerj-11-16290-s004.pdf]
