# Supplementary material for: Body site microbiota of Magellanic and king penguins inhabiting the Strait of Magellan follow species-specific patterns
Source: PeerJ. 2023 Nov 2;11:e16290. doi: 10.7717/peerj.16290 (PMC10625763; doi:10.7717/peerj.16290)
Supplement: Supplemental Information 3 — (A) Psychrobacter sequences in king penguin body sites. (B) Psychrobacter sequences in Magellanic penguin samples. Colors refer to sample type, while number of circles refer to the number of samples where the sequence occurred. [file peerj-11-16290-s003.pdf]

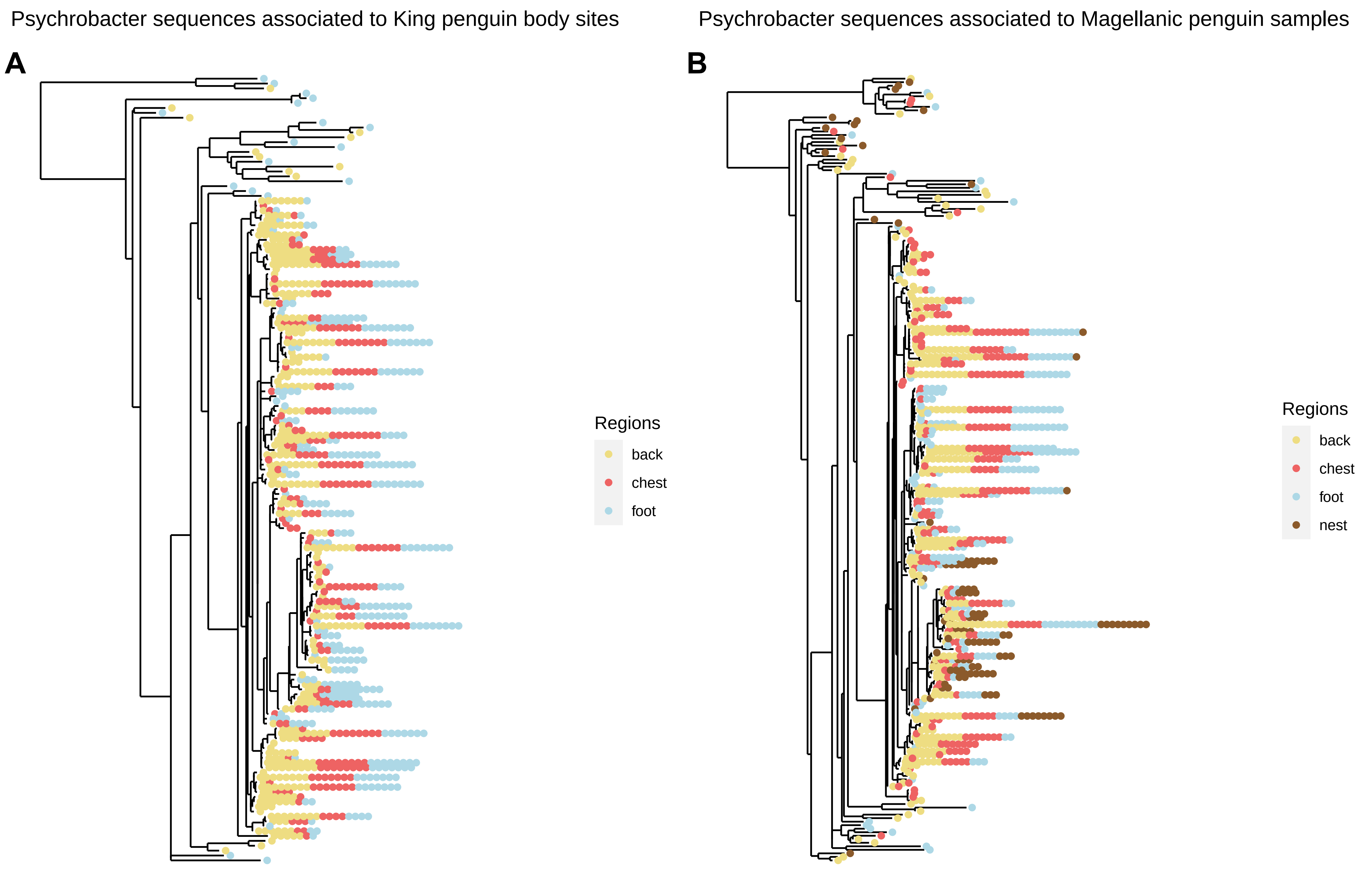

Psychrobacter sequences associated to King penguin body sites
Psychrobacter sequences associated to Magellanic penguin samples
A
B
Regions
back
chest
foot
Regions
back
chest
foot
nest
