# Supplementary material for: Body site microbiota of Magellanic and king penguins inhabiting the Strait of Magellan follow species-specific patterns
Source: PeerJ. 2023 Nov 2;11:e16290. doi: 10.7717/peerj.16290 (PMC10625763; doi:10.7717/peerj.16290)
Supplement: Supplemental Information 5 [file peerj-11-16290-s005.docx]

Supplementary Table 1. Most abundant bacterial genera and proportion of samples where it dominates

| King penguin | | |
| --- | --- | --- |
| Region | Dominant genus | Proportion of samples |
| Chest | *Psychrobacter* | 7/8 |
|  | *Ralstonia* | 1/8 |
| Back | *Psychrobacter* | 6/8 |
|  | *Fusobacterium* | 1/8 |
|  | *Ornithobacterium* | 1/8 |
| Foot | *Psychrobacter* | 6/8 |
|  | *Clostridium sensu stricto 1* | 1/8 |
|  | Unknown | 1/8 |
| Magellan penguin | | |
| Chest | *Psychrobacter* | 7/10 |
|  | Unknown | 2/10 |
|  | *Fusobacterium* | 1/10 |
| Back | Unknown | 5/11 |
|  | *Psychrobacter* | 4/11 |
|  | *Fusobacterium* | 2/11 |
| Foot | *Psychrobacter* | 4/10 |
|  | Unknown | 3/10 |
|  | *Fusobacterium* | 2/10 |
|  | *Staphylococcus* | 1/10 |
| Nest | Unknown | 5/9 |
|  | *Arthrobacter* | 1/9 |
|  | *Clostridium sensu stricto 1* | 1/9 |
|  | *Psychrobacter* | 1/9 |
|  | *Vibrio* | 1/9 |
