# Supplementary material for: Body site microbiota of Magellanic and king penguins inhabiting the Strait of Magellan follow species-specific patterns
Source: PeerJ. 2023 Nov 2;11:e16290. doi: 10.7717/peerj.16290 (PMC10625763; doi:10.7717/peerj.16290)
Supplement: Supplemental Information 6 [file peerj-11-16290-s006.docx]

| King penguin | | | | |
| --- | --- | --- | --- | --- |
| Comparison | Five most influential ASVs | % contribution to difference | % average abundance (a) | % average abundance (b) |
| Back (a) vs foot (b) | ASV19.*Psychrobacter* | 2.018 | 0.057 | 4.009 |
|  | ASV11.*Corynebacterium* | 1.889 | 4.276 | 0.497 |
|  | ASV13.*Psychrobacter* | 1.828 | 4.265 | 0.637 |
|  | ASV8.*Clostridium.sensu.stricto.1* | 1.666 | 0.460 | 3.722 |
|  | ASV1.*Fusobacterium* | 1.537 | 2.620 | 0.727 |
| Back (a) vs chest (b) | ASV2.*Ralstonia* | 3.847 | 2.562 | 0.088 |
|  | ASV3.*Psychrobacter* | 2.046 | 1.923 | 3.073 |
|  | ASV13.*Psychrobacter* | 1.519 | 4.265 | 1.506 |
|  | ASV1.*Fusobacterium* | 1.313 | 2.620 | 0.046 |
|  | ASV11.*Corynebacterium* | 1.198 | 4.276 | 2.475 |
| Foot (a) vs chest (b) | ASV2.*Ralstonia* | 3.842 | 1.803 | 8.808 |
|  | ASV19.*Psychrobacter* | 1.973 | 4.093 | 0.163 |
|  | ASV8.*Clostridium*.*sensu.stricto.1* | 1.682 | 3.722 | 0.409 |
|  | ASV3.*Psychrobacter* | 1.522 | 0.160 | 3.073 |
|  | ASV11.*Corynebacterium* | 1.051 | 0.497 | 2.457 |
| Magellanic penguin | | | | |
| Back (a) vs nest (b) | ASV1.*Fusobacterium* | 4.131 | 8.416 | 1.143 |
|  | ASV5.*Psychrobacter* | 3.632 | 7.268 | 0.003 |
|  | ASV10.f__Micrococcaceae | 2.255 | 1.002 | 5.446 |
|  | ASV30.*Arthrobacter* | 1.494 | 0.148 | 3.064 |
|  | ASV7.*Staphylococcus* | 1.453 | 1.586 | 2.212 |
| Chest (a) vs nest (b) | ASV3.*Psychrobacter* | 4.717 | 0 | 9.435 |
|  | ASV6.*Psychrobacter* | 4.559 | 0 | 9.119 |
|  | ASV4.f__Staphylococcaceae | 4.270 | 0.077 | 8.554 |
|  | ASV1.*Fusobacterium* | 2.875 | 1.143 | 5.461 |
|  | ASV10.f__Micrococcaceae | 2.290 | 5.446 | 1.073 |
| Foot (a) vs nest (b) | ASV1.*Fusobacterium* | 4.922 | 9.790 | 1.143 |
|  | ASV10.f__Micrococcaceae | 2.376 | 0.734 | 5.446 |
|  | ASV9.*Psychrobacter* | 2.354 | 4.709 | 0 |
|  | ASV12.*Psychrobacter* | 2.303 | 4.606 | 0 |
|  | ASV6.*Staphylococcus* | 2.086 | 2.720 | 2.212 |
