# Supplementary material for: Body site microbiota of Magellanic and king penguins inhabiting the Strait of Magellan follow species-specific patterns
Source: PeerJ. 2023 Nov 2;11:e16290. doi: 10.7717/peerj.16290 (PMC10625763; doi:10.7717/peerj.16290)
Supplement: Supplemental Information 7 [file peerj-11-16290-s007.docx]

| Pair comparison | pseudo-F | R^2^ | P.adj |
| --- | --- | --- | --- |
| back vs chest | 1.171 | 0.058 | 0.447 |
| back vs foot | 0.610 | 0.031 | 0.768 |
| back vs nest | 5.895 | 0.246 | 0.004 |
| foot vs chest | 1.053 | 0.055 | 0.447 |
| foot vs nest | 4.115 | 0.194 | 0.003 |
| chest vs nest | 5.001 | 0.227 | 0.003 |
